# Personalized Cardiovascular Disease Prediction and Treatment—A Review of Existing Strategies and Novel Systems Medicine Tools

**DOI:** 10.3389/fphys.2016.00002

**Published:** 2016-01-26

**Authors:** Elias Björnson, Jan Borén, Adil Mardinoglu

**Affiliations:** ^1^Department of Biology and Biological Engineering, Chalmers University of TechnologyGothenburg, Sweden; ^2^Department of Molecular and Clinical Medicine/Wallenberg Laboratory, University of GothenburgGothenburg, Sweden; ^3^Science for Life Laboratory, KTH – Royal Institute of TechnologyStockholm, Sweden

**Keywords:** patient stratification, risk estimation, metabolism, systems medicine, systems biology, network medicine

## Abstract

Cardiovascular disease (CVD) continues to constitute the leading cause of death globally. CVD risk stratification is an essential tool to sort through heterogeneous populations and identify individuals at risk of developing CVD. However, applications of current risk scores have recently been shown to result in considerable misclassification of high-risk subjects. In addition, despite long standing beneficial effects in secondary prevention, current CVD medications have in a primary prevention setting shown modest benefit in terms of increasing life expectancy. A systems biology approach to CVD risk stratification may be employed for improving risk-estimating algorithms through addition of high-throughput derived omics biomarkers. In addition, modeling of personalized benefit-of-treatment may help in guiding choice of intervention. In the area of medicine, realizing that CVD involves perturbations of large complex biological networks, future directions in drug development may involve moving away from a reductionist approach toward a system level approach. Here, we review current CVD risk scores and explore how novel algorithms could help to improve the identification of risk and maximize personalized treatment benefit. We also discuss possible future directions in the development of effective treatment strategies for CVD through the use of genome-scale metabolic models (GEMs) as well as other biological network-based approaches.

## Introduction

Cardiovascular disease (CVD), specifically ischemic heart disease and stroke, remains to be the world leading cause of death by a considerable margin (World Health Organization, 2012). It also remains a challenge to accurately predict who is going to develop CVD. For this purpose, several CVD risk-estimating algorithms including the Framingham risk score (Wilson et al., 1998), Reynolds risk score (Ridker et al., 2007), Pan European score (SCORE; Conroy et al., 2003), ASSIGN Scottish algorithm (Woodward et al., 2007), and QRISK2 UK algorithm (Hippisley-Cox et al., 2008) have been developed (Simmonds and Wald, 2012). The purpose of these algorithms are, by considering traditional risk factors for CVD such as age, BMI, smoking status, and blood lipid parameters (Table 1), to estimate the 10-year risk of a CVD-event so that preventative measures can be initiated for people who will benefit from this intervention. However, the current algorithms have been developed for population-based prediction of CVD and not for personalized prediction, making the task of predicting exactly who is going to develop CVD difficult. For this reason, even though drugs such as statins have shown tremendous benefit in secondary prevention, in a primary prevention setting the benefits have arguably been modest. Preventative intervention is likely beneficial in a subset of the population, hence accurate risk stratification is an essential tool to enable effective preventative treatment. Rapid and continuous efforts are needed to develop novel biomarkers for achieving high diagnostic accuracy to predict CVD.

**Table 1:** **The five CVD risk scores QRISK2, Framingham, ASSIGN, SCORE, and Reynolds include the following parameters**.

**Parameter**	**QRISK2**	**Framingham**	**ASSIGN**	**SCORE**	**Reynolds**
Age	X	X	X	X	X
Smoking status	X	X	X	X	X
Total cholesterol		X	X	X	X
Systolic blood pressure	X	X	X		X
Family history of CVD	X		X		X
HDL cholesterol			X		X
Sex	X		X		
Rheumatoid arthritis	X		X		
Diabetes status	X		X		
Geographic information (postcode)	X		X		
C-reactive protein					X
Cholesterol/HDL ratio	X				
Ethnicity	X				
BMI	X				
Atrial fibrillation	X				
Chronic kidney disease	X				
Blood pressure treatment	X				

*An “X” marks the inclusion of a parameter in the risk score in question*.

Technical breakthroughs have enabled unprecedented progress in the field of omics (i.e., genomics, transcriptomics, proteomics, metabolomics, and lipidomics). Arguably, this should result in great potential in the field of biomarker discovery. Publications in the field of biomarker discovery have increased dramatically over the past two decades, however the increase in the number of clinically useful biomarkers have been meager (Drucker and Krapfenbauer, 2013). In the area of drug development, there is a need for new effective preventative drugs for CVD. But even the most effective drug must be given to the correct subjects. An important distinction must be made between accurate risk identification and accurate personalized prediction of treatment benefit. In a clinical setting, this means that the following two questions should be able to be answered by a CVD risk score as accurately as possible: (i) Will this patient develop CVD within a certain time period? (ii) What is the increase in life expectancy and disease-free years if this particular patient initiates this particular (drug-based or life style-based) intervention? In this review, we discuss the challenges associated with the current CVD risk-estimating algorithms as well as the potential of a systems biology approach to produce better risk scores as well as more effective CVD drugs.

## Current challenges in CVD risk prediction

The ultimate goal of a CVD risk-estimating algorithm is to accurately predict who and when someone is going to develop CVD. This ability should not be confused with the ability of an algorithm to predict how many out of a population will develop CVD during a certain time period. Thus, population-based prediction is different from personalized prediction. In a study by van Staa et al. (2014) this question was addressed by following 1.8 million subjects for an average of 3.3 years. The three widely used risk prediction algorithms Framingham, ASSIGN, and QRISK2 were evaluated to see if the risk scores accurately predicted not only population-based risk but also personalized risk of CVD. To achieve this, the three risk scores were applied at each of the 1.8 million subjects and compared to a competing risk Cox proportional hazard (CRCPH) model. The study reported that the algorithms accurately predicted how many CVD events would occur in the population, and accurately predicted low-risk subjects. However, for high-risk subjects the three algorithms agreed modestly with the CRCPH model. What this study illustrates is that the Framingham, ASSIGN, and QRISK2 CVD risk scores accurately estimate population-based risks and do identify low risk subjects but the algorithms do not accurately predict who is going to develop CVD.

Predicting benefit from an intervention at a personalized level may be a very valuable tool in CVD treatment. Ferket et al. (2012) estimated how much personalized benefit is gained from statin therapy in a population of 2428 Dutch people. A microsimulation model was used to create a personalized calculator of gains in total and CVD-free life expectancy with statin therapy, and the results of the model for each person was compared with the CVD risk predicted by SCORE. The authors observed an average of 0.3 years of increased life expectancy and 0.7 years of increased CVD-free life expectancy gained from an average of 18.3 years of statin therapy. These gains from statin therapy was considered modest, especially considering that side effects were ignored by the model. Further on, statin therapy is currently encouraged with increasing age due to its correlation with higher CVD risk scores. However, importantly; due to competing risk of death from other diseases, it might not follow that increased 10-year risk of CVD implies larger benefit from statin therapy. For example, as stated in the paper “*both a 55-year-old non-smoking woman with a ten-year CVD mortality risk of 2% and a 65-year-old male smoker with a ten-year CVD mortality risk of 15% might both gain one year of CVD-free life expectancy with statin therapy*.” For the entire population, 25% with a low SCORE risk achieved equal or larger gains in CVD-free life expectancy than the median gain in participants with a high SCORE risk estimation. This distinction between risk of CVD and benefit-of-treatment may appear subtle but is important. For secondary prevention, statin therapy have shown tremendous benefit, but what this study illustrates is the challenge of primary prevention treatment decision and that there exist a need for risk scores which also estimates personalized benefit of treatment.

## Current CVD biomarker discovery

With the recent advances in metabolomics technologies, hundreds to thousands of metabolites can be simultaneously detected in tissues and biofluids (e.g., blood and urine) to provide a snapshot of the current physiology. Metabolic signatures of obesity (Newgard et al., 2009), future insulin resistance, T2D (Wang et al., 2011), CVD (Shah et al., 2010; Magnusson et al., 2013), NAFLD, and different types of cancer (Ganti and Weiss, 2011; Tan et al., 2012; McDunn et al., 2013; Zeng et al., 2014) have been characterized for identification of associated risk factors as well as for discovery of novel biomarkers.

Branched chain amino acids (BCAAs), valine, leucine, and isoleucine as well as aromatic amino acids, tyrosine, and phenylalanine were discovered to predict the development of diabetes, which is strongly associated with CVD (Wang et al., 2011). Moreover, BCAAs together with the urea cycle metabolite levels in the plasma were used to predict the development of CVD (Shah et al., 2010). Magnusson et al. (2013) developed a method called diabetes-predictive amino acid (DM-AA) score using the metabolic signature of three amino acids (tyrosine, phenylalanine, and isoleucine) and showed that the plasma level of these amino acids correlated with intima-media thickness, plaque formation and exercise-induced myocardial ischaemia, which are three signs of CVD-related abnormalities. The authors also followed 4577 subjects for an average of 12 years, of which 253 suffered a CVD event. Compared to subjects with lowest quartile values of DM-AA score the odds ratio for CVD development were 1.27, 1.96, and 2.20 for quartile 2, 3, and 4, respectively.

Insulin resistance (IR) has been strongly linked to increased risk of CVD (Ginsberg, 2000), yet no measure of IR is included in the current risk-estimating algorithms (Table 1). The so called *Quantose IR* algorithm has been developed to estimate IR using metabolomics and lipidomics data (Cobb et al., 2013). Quantose IR is apart from the level of fasting insulin based on α-hydroxybutyrate and the two lipid species 1-linoleoylglycerophosphocholine and oleate. This algorithm is an example of a possible improvement in the evaluation of IR through the need of only a fasting blood test and it may increase the accuracy of the current CVD risk-estimating algorithms; however, this has not been systematically evaluated.

Recently, three lipid species TAG(54:2), CE(16:1), and PE(36:5) were discovered as useful for improving the Framingham risk score in 685 subjects of the prospective population-based Bruneck cohort (Stegemann et al., 2014). Addition of another three lipid species and exclusion of HDL-cholesterol and total cholesterol from the Framingham risk score resulted in an additional improvement. Framingham risk score has also been improved by adding the three microRNAs including miR-126, miR-223, and miR-197 as biomarkers of CVD (Zampetaki and Mayr, 2012). Moreover, Bolton et al. (2013) evaluated a panel of 27 single nucleotide polymorphisms (SNPs), discovered from genome-wide association studies, to predict the occurrence of coronary heart disease. Compared to a Cox proportional hazard model based on traditional risk factors, the addition of the SNP panel significantly improved the accuracy of the model. Hence, evident improvements upon the traditional risk scores estimated by the existing algorithms have already been achieved by omics-derived biomarkers of CVD. However, the gains are arguably modest.

## Why have so few new biomarkers been discovered?

There exist a large discrepancy between the number of biomarker discovery publications and the number of new biomarker patents (Drucker and Krapfenbauer, 2013). For all diseases (not only CVD) only 1–2 new biomarkers were approved by the Food and Drug Administration each year in the US between 1995 and 2009 despite the enormous technical advances in the omics fields during the same period (Anderson, 2010). There are probably a number of reasons for this, including lack of standardized biomarker discovery pipeline, lack of good verification platform for large sample sets and lack of an underlying theory of biomarkers.

There are three categories in which newly discovered potential biomarkers fall into: *chance, bias*, and *generalizability*. The only category that may result in a potentially clinically useful biomarker is the latter. The risk of a false discovery increases with increasing number of measured parameters. Therefore, the current ability to measure hundreds to thousands of analytes in a single experiment will result in potential false discoveries. However, this problem can be remedied by commonly used statistical techniques and is therefore probably not the largest explanation to the lack of novel biomarkers.

The issue of *bias* is however not a problem to be overcome by statistical analysis techniques but is instead inherent in the experimental design. For example, when a biomarker study is commenced a study population is separated into a diseased group and a control group. However, when analyzing the characteristics of the groups, it might be discovered that the diseased group is also in average older and heavier than the control group. Is it then possible to say that a discovered biomarker is a biomarker of the disease, the age, or the weight? For this reason, the groups are often matched against each other to minimize known confounding factors. However, unknown confounding factors might still bias the study. The only remedy to this problem is randomization. Unfortunately, by definition, a biomarker discovery study can never be randomized thus making the risk of so called *bias of inequality at baseline* an inherent problem of biomarker discovery. How important this issue is and if it can explain the lack of accurate CVD biomarkers is currently unknown, but it is likely an important contributing factor. Bias can also be introduced if the samples from the different groups are treated differently throughout the analysis pipeline. It is therefore of vital importance that the handling and analysis of samples are conducted consistently. If there is bias of inequality at baseline between two groups, there is a risk that a measured parameter will correlate with an unknown confounding factor and not with the disease. The risk to have *any* discovery due to bias thus increases both with the number of confounding factors *and* with the number of parameters analyzed. To overcome this problem it might (paradoxically to the field of omics) be desirable to measure as *few* parameters as possible. Thus, one way of achieving maximum chance of detecting true biomarkers is to have a biomarker theory. An underlying theory would be able to *a priori* point to what should be measured, thus limiting the need to measure lots of parameters.

As an alternative to the search for a *single* biomarker of CVD, another approach is to use a panel of biomarkers. If such a panel is to be highly sensitive and highly specific it requires that the individual biomarkers are so called *orthogonal* against each other. This means that every biomarker adds diagnostic value to the panel rather than just co-vary with other markers. Recent technologies such as protein multiplex platforms do invoke hope that effective biomarker-panels of CVD could be created and used in the clinic.

## Novel tools in systems medicine

Genome Scale Metabolic Models (GEMs) are employed for simulating the metabolism of cells/tissues. When generating a GEM, all known metabolic reactions in a particular cell or tissue are integrated into one network topology. Once the model has been constructed, it can be used in conjunction with flux balance analysis which allows for *in silico* metabolic simulation of the cell or tissue type in question (Mardinoglu and Nielsen, 2012, 2015; Mardinoglu et al., 2013; O'Brien et al., 2015; Yizhak et al., 2015). GEMs in combination with transcriptomics, proteomics, metabolomics, or lipidomics data have the potential to identify perturbed metabolic subnetworks *in silico* (Agren et al., 2012, 2014; Shoaie et al., 2013, 2015; Yizhak et al., 2013, 2014a,b; Galhardo et al., 2014; Mardinoglu et al., 2014; Gatto et al., 2015; Ghaffari et al., 2015; Varemo et al., 2015; Zhang et al., 2015). GEMs constitute a possible powerful tool in the area of human complex disease since it enables the potential of pathophysiological understanding of a disease (Ryu et al., 2015).

Another interesting tool in systems medicine is protein–protein interaction (PPI) networks (Rolland et al., 2014). PPI networks has the potential to provide useful information in CVD, since each protein is placed in a larger network context and thus alterations in proteins in the diseased state can be compiled and translated into meaningful biological tasks. For example, if 100 different proteins are shown to be altered in the blood macrophages or endothelial cells of people with CVD and 80 of them happen to be highly connected, shown by a PPI, then that part of the network and the related metabolic function could be concluded to be perturbed in the diseased state. Further on, if a few of the proteins are shown to interact with lots of the other disease-related proteins, these highly connected proteins might be central to the disease progress itself. Thus, PPIs could identify central hubs in the disease-network, hubs that might provide pathophysiological understanding and be suitable as drug targets.

As mentioned, an *a priori* theory of biomarkers could aid in biomarker discovery. A theory of biomarkers could be created through the use of GEMs and PPI networks. A hypothetical example for use of GEMs in CVD would be to model the metabolism of cell types in the blood, for example macrophages, endothelial cells or myocardial cells. If this would be done, predictions about the metabolism of these cells and possible metabolic alterations in CVD could be enabled. Specifically, if GEMs would provide a mechanistic understanding of for example macrophages and their possible metabolic alterations in CVD, a limited set of plausible biomarkers (proteins or metabolites) could be selected and measured independently in a biomarker discovery study. This approach, coupled with stringent experimental biomarker discovery design would limit the risk of bias and could increase the chance of discovering clinically useful biomarkers.

A concrete example for using GEMs which could be relevant to CVD involves macrophage activation. Since there is a link between inflammation and CVD and since macrophages play an important role in the build-up of atherosclerotic plaques, studying the metabolism of macrophages could aid in the understanding of CVD. Bordbar et al. (2012) used genome-scale metabolic modeling in combination with transcriptomics, proteomics, and metabolomics to reveal the metabolic features and modulators of macrophage activation. They identified metabolites which enhanced (glucose and arginine) and suppressed (tryptophan and vitamin D3) macrophage activation. These particular metabolites were previously known to be associated with immunoactivation but the mechanism was unknown. Such a mechanistic insight into what regulates macrophages could help in designing effective interventions. In this case, the plausible intervention would be to limit glucose and arginine intake and increase tryptophan and vitamin D3 intake to decrease the activity of the blood-macrophages. Probably, an intervention like this is not as straight-forward but it does provide a rational approach for the development of treatment strategies which could be tested empirically.

Heart performance is naturally relevant for cardiovascular health and is plausibly affected by the heart's energy metabolism. Little is however known about the energy metabolism of the heart in humans *in vivo*, during varying nutrient conditions and pathological conditions such as heart failure and diabetes. In order to simulate cardiac performance Karlstädt et al. (2012) developed *CardioNet*—a GEM covering the metabolism of the cardiomyocyte. Simulations for different nutrient conditions were performed and the efficiency (how much ATP was produced compared to substrate and oxygen consumption) of the heart was evaluated. Differences were seen when comparing different combinations of substrates in terms of cardiac output. The authors observations suggested that high levels of the ketone body acetoacetate (which can be seen in for example diabetes) would decrease cardiac output and increase ROS production indicating possible decreased cardiac contractility. It is currently not known how e.g., diabetes could affect cardiac health. The study by Karlstädt et al. provide a possible pathophysiologic mechanisms of heart malfunction related to diabetes and more generally provide a framework for evaluating how varying oxygen and nutrient conditions could affect the heart.

In order to simulate the entire human cellular and tissue functions in a holistic approach, a whole cell/tissue model could be used. One example of a whole-cell model of the human pathogen *Mycoplasma genitalium* has been successfully developed and simulation of dynamic cellular states has been demonstrated (Karr et al., 2012). This holistic approach has not yet been employed on human cells but does show the potential use of such models. This process typically involves construction and employment of metabolic, regulatory, signaling, and PPI networks in conjunction with GEMs. The COBRA Toolbox (Schellenberger et al., 2011) and RAVEN Toolbox (Agren et al., 2013) which are valuable supports for researchers in genome-scale metabolic modeling should also be expanded to deal with simulation of these integrative models. Considering the 3675 protein coding genes (18% of the genome) in the generic human GEM HMR2 (Mardinoglu et al., 2014) and their interactions with other proteins in biological networks, such integrated computational models may provide further information about the relationship between the genotype and phenotype of CVD.

There are a number of hurdles to overcome for successful simulation of human metabolism in a biologically relevant matter. Reconstructing GEMs involves correctly defining, for each metabolic reaction, the stoichiometry, the substrate(s), the product(s), the enzyme(s), and the gene(s) which characterize that specific reaction. This information has to be correct for thousands of reactions. During the generation of the GEMs, the network often needs to be so called gap-filled in order for the network to be connected and complete. This gap-filling step is one source of potential errors in the model. Compartmentalization of the reactions is also a relevant issue, not least when constructing human GEMs. It is often not known where a reaction occurs in the cell and whether the substrate/product can be exported/imported into other compartments. Even though there is an extensive effort in defining the subcellular localization of proteins (Kampf et al., 2014; Uhlén et al., 2015), the complete draft information will not be available for another few years.

Another issue relevant for human cell specific metabolic models regards defining the environment. In microbial conditions, the growth media is very well-defined so that the possible uptake and secretion fluxes are also known. For human cells the environment is much less known, which can greatly affect the behavior of the model. For a GEM to simulate the function of a cell/tissue accurately a so called objective function needs to be defined. Usually, maximization of growth is used as an objective function for microorganisms. However, defining an objective function for human cells is not as straight-forward. For human cells, this could feasibly be very context specific, depending on for example regulation and signaling effects. Integrating regulatory and signaling networks with GEMs could therefore be important in order to capture biologically relevant behavior. This integration is however a challenging task due to increase in size of the networks. A GEM usually needs a pre-defined biomass equation. The biomass equation greatly influences the behavior of the model (directs the fluxes) and thus the model is very sensitive to the definition of the biomass equation. A number of issues has been raised on this topic and the genome-scale metabolic modeling community has responded successfully (Chindelevitch et al., 2015; Ebrahim et al., 2015).

Lastly, a model is often validated by its predictive ability, for example for a microorganism GEM to predict the growth rate and production rate of various substances. However, models are rarely shown to not be able to perform infeasible tasks. The unknowns in cell biology coupled with the degrees of freedom in the generated networks makes genome scale modeling challenging. However, several cancer related studies, testifying to the value of the genome scale modeling in portraying a network-level view of the cancer metabolism and in discovery of novel drug targets and biomarkers have been recently reviewed (Yizhak et al., 2015) and a similar framework could plausibly be used for CVD.

**Figure 1:**
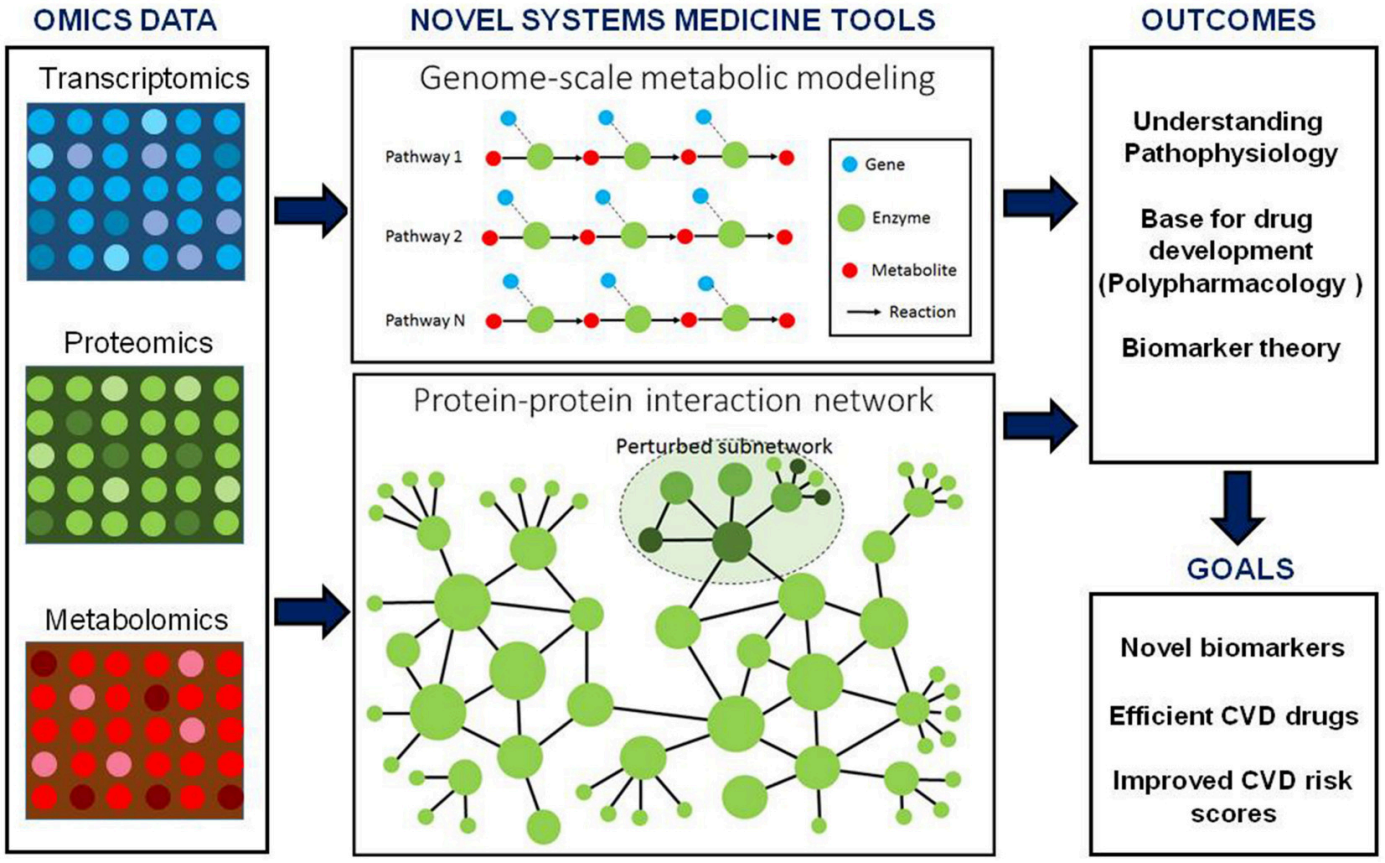
**Integration of genome-scale metabolic models and other biological networks including protein–protein interactions may provide an excellent scaffold for integration of omics data including transcriptomics, proteomics and metabolomics data**. These integrated models can be used for the discovery of biomarker and identification of drug targets. Moreover, biomarkers predicted for CVD can be used together with other risk estimating algorithms for personalized risk prediction of CVD.

In conclusion, placing high-dimensional omics data in a network context, whether through the use of GEMs, PPIs, or other networks (e.g., regulatory and signaling), may allow for an increased pathophysiologic understanding of CVD. In addition, GEMs together with other networks could provide a rational approach to biomarker discovery, limiting the risk of bias and increasing the chance of improving CVD risk scores (Figure 1). However, important limitations do currently exist regarding the biological relevance of human GEMs.

## Network medicine and drug development

As stated, network-dependent analyses may allow for identification of metabolic perturbations in CVD. Biological networks have arguably evolved to be robust. For example, single blockade of 85–90% of all proteins in yeast do not result in any noticeable phenotypic alterations (Peters, 2013). Similarly, knock-out studies in mice suggest that only 10% of all potential drug target genes would have any effect as single targets (Peters, 2013). In the traditional reductionist approach to drug development, a disease modifying activity is reduced to a single target. While this can be effective for certain diseases, it may not be enough for treatment of a complex disease such as CVD. CVD specifically could have multiple or complex causes which result in network-level perturbations. If this is the case, an alternative approach to CVD drug development would be identification of network-level perturbations and developing drugs that can affect the network rather than only a single protein.

The upcoming branch of network medicine or *polypharmacology*, integrates systems biology tools with pharmacology. Recently, a drug-target and a target–target interaction network was constructed to identify which targets of CVD drugs that possesses the most interconnectedness with drugs and other targets (Zheng et al., 2014). These targets have high probability of being important hubs in the CVD-related metabolic networks and thus interesting to treat with a multi-target compound. Subsequent virtual screening of compounds revealed several potential multi-target drug candidates and *in vitro* validation of five randomly selected candidate compounds revealed that four of them could indeed bind to these targets and thus possibly affect the CVD-related metabolic network. However, this approach to drug discovery could perhaps also increase the risk of adverse effects precisely because the compounds in question are unspecific. Nevertheless, this method illustrates how a polypharmacological approach to CVD drug development could be conducted. If these types of methods of drug development will produce effective CVD-risk lowering interventions remains however to be seen.

Risk scores based on multi-biomarker panels might also aid in system-level drug development. If a potential drug affects a single target but does not affect a plethora of other biomarkers, this could provide an early indicator that the drug candidate might not prevent CVD. However, if multiple markers change after administration of a potential drug candidate, that might be indicative of reduced risk of CVD and a potentially successful drug. Risk scores based on multi-biomarker panels could of course similarly be used for evaluation of other types of interventions such as diet, and not only drug-based interventions. The field of polypharmacology is, albeit promising, still new. Future efforts in this area could hopefully result in the development of novel preventative CVD medications.

## Conclusions

Systems medicine uses omics data for reconstruction of cellular networks. High dimensional omics data is often not easy to directly translate into biological meaning. Therefore, the systems medicine approach could, by integrating different kinds of omics data and putting them in a network context, enable pathophysiological understanding of a disease in question. Systems medicine aims at identifying how the integrated *network*, rather than single genes or proteins, is altered in a diseased state. This approach allows for identification of perturbed subnetworks and may, apart from providing pathophysiologic understanding of the disease, also create a base to predict biomarkers and identify subnetworks as drug targets. This information could lead to more accurate CVD risk scores as well as more effective drugs/interventions.

In conclusion, it is important for each patient to understand his/her own risk of CVD as well as likely benefit of treatment to weigh against any potential side effects, thus there is a need for accurate personalized risk scores in conjunction with personalized prediction of treatment benefit. As illustrated, current risk-estimating algorithms can in this setting be improved upon. Accurate risk scores, more effective drugs and personalized estimation of benefit from treatment are three much needed tools in the area of CVD prevention. A systems medicine approach can hopefully provide value in all these areas.

## Author contributions

All three authors actively contributed in writing and editing of the manuscript.

### Conflict of interest statement

The authors declare that the research was conducted in the absence of any commercial or financial relationships that could be construed as a potential conflict of interest.
